# Attention-Gated Deep-Learning–Based Automatic Digitization of Interstitial Needles in High-Dose-Rate Brachytherapy for Cervical Cancer

**DOI:** 10.1016/j.adro.2023.101340

**Published:** 2023-08-10

**Authors:** Yuenan Wang, Wanwei Jian, Lin Zhu, Chunya Cai, Bailin Zhang, Xuetao Wang

**Affiliations:** a Department of Therapeutic Radiology, Yale University School of Medicine, New Haven, Connecticut; b Department of Radiation Therapy, The Second Affiliated Hospital, Guangzhou University of Chinese Medicine, Guangzhou, China

## Abstract

**Purpose:**

Deep learning can be used to automatically digitize interstitial needles in high-dose-rate (HDR) brachytherapy for patients with cervical cancer. The aim of this study was to design a novel attention-gated deep-learning model, which may further improve the accuracy of and better differentiate needles.

**Methods and Materials:**

Seventeen patients with cervical cancer with 56 computed tomography–based interstitial HDR brachytherapy plans from the local hospital were retrospectively chosen with the local institutional review board's approval. Among them, 50 plans were randomly selected as the training set and the rest as the validation set. Spatial and channel attention gates (AGs) were added to 3-dimensional convolutional neural networks (CNNs) to highlight needle features and suppress irrelevant regions; this was supposed to facilitate convergence and improve accuracy of automatic needle digitization. Subsequently, the automatically digitized needles were exported to the Oncentra treatment planning system (Elekta Solutions AB, Stockholm, Sweden) for dose evaluation. The geometric and dosimetric accuracy of automatic needle digitization was compared among 3 methods: (1) clinically approved plans with manual needle digitization (ground truth); (2) the conventional deep-learning (CNN) method; and (3) the attention-added deep-learning (CNN + AG) method, in terms of the Dice similarity coefficient (DSC), tip and shaft positioning errors, dose distribution in the high-risk clinical target volume (HR-CTV), organs at risk, and so on.

**Results:**

The attention-gated CNN model was superior to CNN without AGs, with a greater DSC (approximately 94% for CNN + AG vs 89% for CNN). The needle tip and shaft errors of the CNN + AG method (1.1 mm and 1.8 mm, respectively) were also much smaller than those of the CNN method (2.0 mm and 3.3 mm, respectively). Finally, the dose difference for the HR-CTV D90 using the CNN + AG method was much more accurate than that using CNN (0.4% and 1.7%, respectively).

**Conclusions:**

The attention-added deep-learning model was successfully implemented for automatic needle digitization in HDR brachytherapy, with clinically acceptable geometric and dosimetric accuracy. Compared with conventional deep-learning neural networks, attention-gated deep learning may have superior performance and great clinical potential.

## Introduction

Cervical cancer is the most common cause of cancer death among women.^1^ Despite being a highly preventable and treatable cancer, an estimated 342,000 people worldwide died of the disease in 2020. High-dose-rate (HDR) brachytherapy after external beam radiation therapy is the standard-of-care treatment for patients with cervical cancer.^2^ Computed tomography (CT)–based HDR brachytherapy is often involved with freehand insertion of interstitial needles, multineedle digitization, and treatment planning for precision radiation therapy.

The dose distribution of HDR brachytherapy for patients with cervical cancer often depends on the accuracy of needle digitization, where a small amount of uncertainty in needle rotation or tip position may affect the dwell time and position.^3^ However, the manual digitization used in the current practice of HDR brachytherapy can be time consuming, user dependent, and error prone. There is an urgent need to implement automatic needle digitization integrated with current treatment planning systems for HDR brachytherapy.

There are challenges in automatic digitization of interstitial needles. First, the geometric relationship of needles can be complex, with crossing or touching needles impeding accurate and reliable needle digitization. Second, the metal artifacts of interstitial needles can be severe in CT simulation and prevent precise automatic needle digitization.^4^ Consequently, accurate and precise needle digitization independent of planners can be technically difficult.

Several methods of automatic needle or applicator digitization have been proposed previously.^4^, ^5^, ^6^, ^7^, ^8^, ^9^, ^10^, ^11^, ^12^, ^13^ However, most of them were focused on digital reconstructions of  tandem & ovids applicator (T&O) or vaginal cylinder applicators. Zhou et al^5^ introduced a web-based AutoBrachy system for vaginal cylinder applicators. Deufel et al^6^ used Housfield units thresholding and a density-based clustering algorithm for T&O with treatment planning evaluation. Recently, deep-learning–based solutions have attracted increasing attention owing to their wide application to medical image analysis.^14^ For example, deep convolutional neural networks (CNNs), such as Unet, have achieved significant progress in the past few years, including image segmentation,^15^ image synthesis,^16^ dose prediction,^17^^,^^18^ and lesion detection.^19^ For automatic digitization of needles in brachytherapy, deep-learning–based techniques have been used in different image modalities.^4^^,^^7^, ^8^, ^9^, ^10^ Zaffino et al^7^ adopted a 3-dimensional (3D) Unet model for segmentation of multiple catheters in intraoperative magnetic resonance imaging (MRI). Zhang et al^9^ presented a 3D Unet model incorporating spatial attention gates and total variation regularization for needle localization in ultrasound-guided HDR prostate brachytherapy. Jung et al^4^ extended their AutoBrachy system with a 2.5-dimensional Unet model to digitize the interstitial needles in 3D CT images for HDR brachytherapy. Other attempts to automatically segment and digitize applicators based on 3D Unet in CT images were also reported,^11^, ^12^, ^13^ but no attention-added CNN for needle digitization has been implemented thus far.

The standard Unet CNN structure uses the skip connection at multiscale levels, which leads to relearning the redundant low-level features.^20^ The redundancy may slow down the convergence or reduce the accuracy of tasks. We aimed to propose an attention-integrated 3D Unet CNN method to highlight salient regions and suppress irrelevant information in needle digitization. Because the additive attention gates (AGs) may increase model computation,^20^ we proposed to apply group normalization^21^ to improve computation efficiency. The automatic needle digitization was subsequently compared in terms of geometric and dosimetric accuracies. Compared with the standard 3D Unet method, the incorporation of spatial AGs may increase the computation intensively.^20^ We proposed to integrate group normalization^21^ at the encoder to improve the convergence speed, which may be especially suitable for a small batch size such as that in our study.

We aim to implement an attention-gated deep-learning model for automatic needle digitization of HDR brachytherapy planning, which may improve efficacy and decrease heterogeneity introduced by manual digitization from different planners in the current practice of HDR brachytherapy.

## Methods and Materials

The planning workflow of automatic needle digitization in HDR brachytherapy included 2 main steps: (1) regions of interstitial needles nearby were segmented via 3D Unet, and (2) needle trajectories were digitized as channels for the HDR radioactive source incorporated in the treatment planning process.

### Patient selection

Seventeen patients with different stages of cervical cancer who underwent freehand interstitial needle insertions in HDR brachytherapy were retrospectively selected. The patients’ characteristics are shown in Table 1. This study was approved by the local institutional review board. Each patient was treated with 4 or 5 fractions of HDR brachytherapy with 4 to 6 trocar stainless steel needles inserted during each fraction delivered on the Flexitron HDR treatment unit (Elekta AB, Stockholm, Sweden). There were 56 CT sets acquired on the CT simulator (Siemens SOMATOM Sensation Open, Siemens Medical System, Germany) with 5-mm slice thickness, a resolution of 512 × 512, and 0.8 mm in-plane voxel size (range, 0.6-1.0 mm). All interstitial needles were trocar with a diameter of 1.5 mm and a length of 200 mm (Elekta AB). All HDR brachytherapy plans were created on the Oncentra Brachy Planning System, version 4.6 (Elekta AB), by experienced medical physicists and approved clinically by experienced radiation oncologists.

**Table 1:** Patient characteristics

Characteristic	Patients (N = 17)
Age, mean, y	53.6 ± 11.0
Volume of HR-CTV, mean, cm^3^	122.8 ± 67.6
Prescription dose per fraction, Gy	6 Gy × 4-5 fractions
Number of needles, mean ± SD	4.0 ± 0.6
FIGO clinical stage, no. of patients	
I A	4
II A	1
II B	4
III A	1
III B	2
III C	5
Total plans	56
CT slice thickness, mm	5
CT voxel average dimension, mm	0.8 × 0.8

*Abbreviations:* CT = computed tomography; FIGO = International Federation of Gynecology and Obstetrics; HR-CTV = high-risk clinical target volume.

### Image preprocessing

Fifty planning CT sets were randomly selected as the training data and the remaining 6 as the validation data. The mask images of manually digitized needles were considered as ground truth when the 3D Unet and attention-gated 3D Unet were trained separately. We applied image augmentation, including rotation, horizontal flip, vertical flip, and scaling on the CT images and the corresponding needle-mask images, to prevent overfitting on training a relatively small data set. We also cropped the image size to 256 × 256 to improve computational efficiency, as routinely done in the preprocessing of images before training the deep-learning models.^6^

### Deep-learning neural networks with and without AGs

#### 3D Unet (CNN)

**Figure 1:**
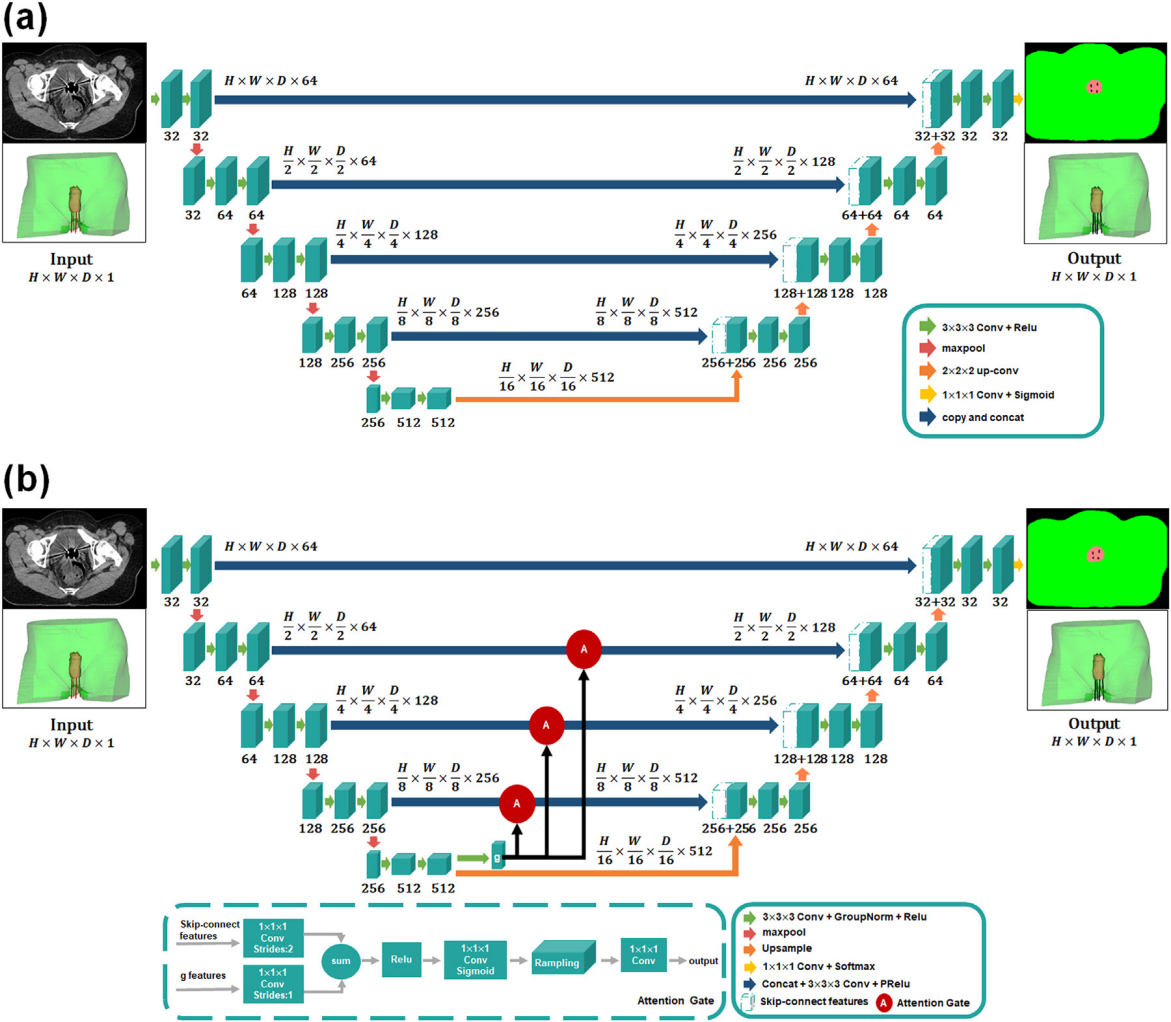
The network architecture of (A) 3-dimensional (3D) Unet and (B) attention-gated 3D Unet. The red-circled A represents the attention gate incorporated in the standard convolutional neural network (3D Unet).

The Unet model, a type of CNN in deep-learning algorithms, has been widely used in image segmentation and radiation therapy planning.^22^, ^23^, ^24^, ^25^ The network architecture of a traditional 3D Unet in our study is illustrated in Fig. 1A. The encoding part consisted of 2 convolutional layers with a kernel size of 3 × 3 × 3, followed by rectified linear unit (Relu) and max-pooling operation. The coarse-feature maps were extracted at multiple scales in the encoding stage and later combined with fine-feature maps in the decoding stage through skip connections. The decoding part consisted of an up-convolution with a stride of 2 followed by Relu and concatenation, except the final layer as the 1 × 1 × 1 convolution with the sigmoid activation. The skip-connections trick combined the coarse- and fine-level feature maps to obtain more refined structures. There were a total of 22 convolutional layers with zero padding in each layer in the proposed 3D Unet. Inspired by Kearney et al,^20^ the entire 3D Unet model was trained with a soft Dice similarity coefficient (DSC) loss function, which is described in Eq. 3.

#### 3D Unet with attention gates

Attention gates were incorporated into the 3D Unet model previously described to highlight salient features for small needles and progressively suppress feature responses in irrelevant regions. To reduce false-positive errors in needle digitization, we integrated AGs before the skip connections.^26^ The multiscale coarse features extracted from the encoder were selected to incorporate into AGs before skip connections to merge relevant features of fine needles with deep-learning neural networks. Attention gates in the CNN + AG method had weighted relevant spatial features (Fig. 1B) and were formulated as follows:

(1) $$q_{\text{att},\;i}^{l}=\psi^{T}\left(\sigma_{1}\left(W_{x}^{T}x_{i}^{l}+W_{g}^{T}g\;+b_{\text{xg}}\right)\right)+b_{\psi }$$

(2) $$\alpha^{l}=\sigma_{2}\left(q_{\text{att}}^{l}\left(x^{l},g;\Theta_{\text{att}}\right)\right)$$

where $\sigma_{1}\left(\cdot \right)$ was the Relu and $\sigma_{2}\left(\cdot \right)$ was a sigmoid activation function to restrict the range of attention coefficients. The gating signal *g* was the coarse-scale activation map to encode features from large spatial regions, and *x* represented the fine-level feature map. Attention gates were characterized by the parameters $\Theta_{\text{att}}$, which included the 1 × 1 × 1 convolution ($W_{x}^{T},$ $W_{g}^{T}$, $\psi^{T}$) and bias terms ($b_{\psi },\;b_{\text{xg}}$), and all parameters of AGs could be updated with the standard back-propagation. The trilinear interpolation was used for grid resampling of attention coefficients. Therefore, the CNN + AG model was designed to focus on target regions from a wide range of image foreground content.

#### Training and validation of deep-learning models

The 3D Unet, both with and without AGs, was trained with a soft Dice loss function to mitigate the imbalance objectives during the needle's digitization:

(3) $$L_{\text{SoftDice}}=\;1-\frac{2\left|P\cap T\right|}{\left|P\right|+\left|T\right|+\left|\varepsilon \right|}$$

where *P* and *T* represented the prediction and ground truth, respectively.εwas a constant value of 0.0001, which was introduced to prevent the numerator from being divided by 0.

We used adaptive moment estimation (Adam) optimizer with a learning rate of 5 × 10^–4^ to train deep-learning networks. The maximum epoch was set to 200, with a batch size of 1. The training stage was undertaken on an Intel Core i9-10980XE CPU @3.00GHz, NVIDIA Quadro RTX 5000 with 16 GB of memory. Both models were implemented on the PyTorch framework.

#### Needle digitization and dose calculation

After acquiring the segmented contours, we calculated the central trajectory coordinates of each needle by an open-source software 3D Slicer (Slicer 4.10.2). Moreover, we performed polynomial curve fitting to avoid a systematic error.

The central trajectory coordinates of each needle generated by the deep-learning–based method were rewritten into the original treatment plan file. The properties of the treatment plan, such as prescription dose, number of dwell, dwell time of source, step size of source, needle length, and needle offset, were kept the same. Dose recalculation was conducted using the Oncentra Brachy treatment planning system (TPS), version 4.6 (Elekta AB), with the same parameters. Dose-volume histograms (DVHs) and 3D dose distributions were compared between manual digitization and automatic digitization of interstitial needles using CNN and CNN + AG methods.

### Evaluation

#### Geometric evaluation: Needle digitization

The distance metrics, including the DSC, Jaccard index (JI), and Hausdorff distance (HD)^27^ were used to assess accuracy. The DSC metric measured the spatial overlap between the prediction and ground truth regions:

(4) $$\text{DSC}\;=\frac{2\times P\cap T}{P+T}$$

The JI measured the similarity between the 2 regions by calculating the ratio of intersection and union as

(5) $$\text{JI}=\frac{P\cap T}{P\cup T}$$

where *P* and *T* are the deep-learning model prediction and ground truth mask regions, respectively. The HD was defined as

(6) HD(A,B)=max(D(A,B),D(B,A))

(7) $$D\left(A,B\right)=\text{ma}x_{a\in A}\text{mi}n_{b\in B}\left|\left|a-b\right|\right|$$

where *A* and *B* are the measured voxel set of deep-learning model prediction and ground truth; *a* and *b* are the points of sets *A* and *B*, respectively; and ||·|| is the Euclidean distance between points A and B. The HD metric measures the maximum mismatch between the automatic segmentation and ground truth. A smaller HD and larger DSC and JI coefficients indicate better segmentation performance.

In terms of the geometric evaluation of needles central trajectory, we used the tip error and shaft error^7^ to evaluate the accuracy of needle position. The needle tip error was defined as

(8) $$E_{\text{tip}}=\frac{1}{N}\sum_{I\;=\;1}^{N}\left|\;P_{i}-I\;\right|$$

where *N* indicated the total needles path number. *P_i_* and *T_i_* were the predicted length and ground truth length for the *i*th needle. The needle shaft error was defined as

(9) $$E_{sh\text{aft}}=\frac{1}{\text{MN}}\sum_{j\;=\;1}^{M}\sum_{I\;=\;1}^{N}\left|\left|\;P_{\left(x,\;y\right)}-T_{\left(x,\;y\right)}\;\right|\right|$$

where *M* indicated the number of measured points in the needle's central trajectories, *P*_(_*_x_*_,_*_y_*_)_ represented the predicted coordinates, and *T*_(_*_x_*_,_*_y_*_)_ represented the ground truth coordinates for the *i*th needle. We performed paired *t* tests to assess whether the geometric results between CNN and CNN + AG methods were statistically significant at *P* < .05.

#### Dosimetric evaluation

The dose recalculation of deep-learning models in the Oncentra Brachy TPS was the same as that of manual digitized needles based on the updated American Association of Physicists in Medicine Task Group Report 43.^28^ The isodose lines and DVHs were compared between automated and manual needle digitization. The DVH metrics, such as D_90%_ and D_100%_ for the high-risk clinical target volume (HR-CTV) and D_2 cc_ for organs at risk (OARs)—the bladder wall, rectum wall, intestines, and sigmoids—were reported.^29^ In general, the dosimetric differences between the manual and automatic plan were assessed as follows:

(10) $$\text{Dose}\;\text{difference}\;=D_{\text{manual}}-D_{\text{automatic}}$$

(11) $$\text{Relative}\;\text{dose}\;\text{difference}\;=\frac{D_{\text{manual}}-D_{\text{automatic}}}{D_{\text{manual}}}$$

## Results

### Geometric comparison

The number of interstitial needles was 4 to 6 in the training set and 3 to 6 in the validation set. The average DSC and JI of CNN + AG were 93.7% and 88.2%, respectively (Table 2), demonstrating consistency with the ground truth of manual needle digitization using deep learning. Furthermore, the mean DSC and JI were statistically significantly higher (*P* < .05) using CNN + AG than using CNN only. The average HD obtained using CNN + AG was 2.9 mm smaller than that using CNN only, showing deep learning with AGs was more accurate than CNN only. The average difference of tip and shaft positions using CNN + AG versus ground truth of manual segmentation was 1.1 ± 0.7 mm and 1.8 ± 1.6 mm, respectively, which was also more accurate than the CNN-only method. Our proposed CNN + AG method was superior to the CNN method, revealing that the integrated attention mechanism with group normalization of the deep-learning model was feasible in needle digitization, despite needles crossing or touching in the CT of freehand needle insertions in HDR brachytherapy.

**Table 2:** Needle digitization for 6 validation cases

	DSC, %	JI, %	HD, mm	Tip error, mm	Shaft error, mm	Time, s
3D CNN	88.5 ± 1.8	79.4 ± 2.8	5.8 ± 3.9	2.0 ± 1.6	3.3 ± 3.3	1.3
3D CNN + AG	93.7 ± 1.4	88.2 ± 2.5	3.0 ± 1.9	1.1 ± 0.7	1.8 ± 1.6	1.6
*P* value	<.05	<.05	-	-	-	-

*Abbreviations:* 3D CNN = 3-dimensional convolutional neural network; AG = attention gate; DSC = Dice similarity coefficient; HD = Hausdorff distance; JI = Jaccard index.

**Figure 2:**
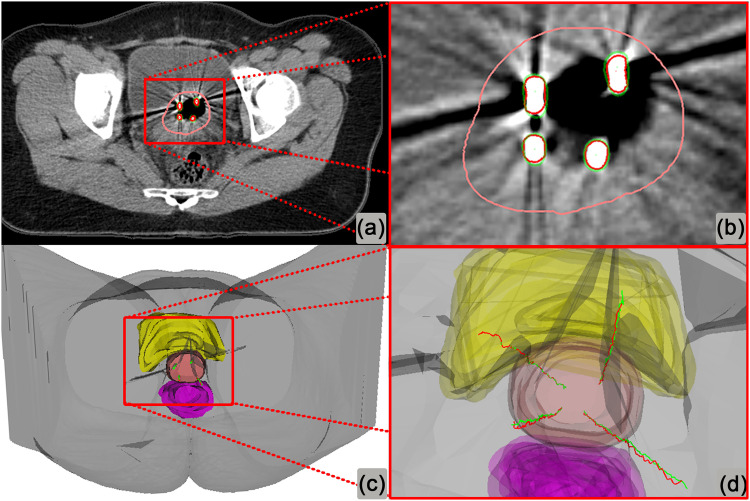
The needle digitization of a patient example using attention-gated 3-dimensional (3D) Unet. Red indicates manual contours and central trajectories of needles; green, automatic needle digitization using attention-gated 3D Unet; Pink, high-risk clinical target volume; yellow: bladder; and purple: rectum.

One example of needle digitization generated by attention-gated 3D Unet is shown in Fig. 2. The digitization using attention-gated 3D Unet was in good agreement with the ground truth. The total time of training for the proposed attention-gated 3D Unet versus 3D Unet only was about 12.6 and 3.3 hours, respectively. However, in the validation set, the time of automatic needle digitization was only 1 to 2 seconds per patient, without a significant difference.

### Dosimetric analysis

**Figure 3:**
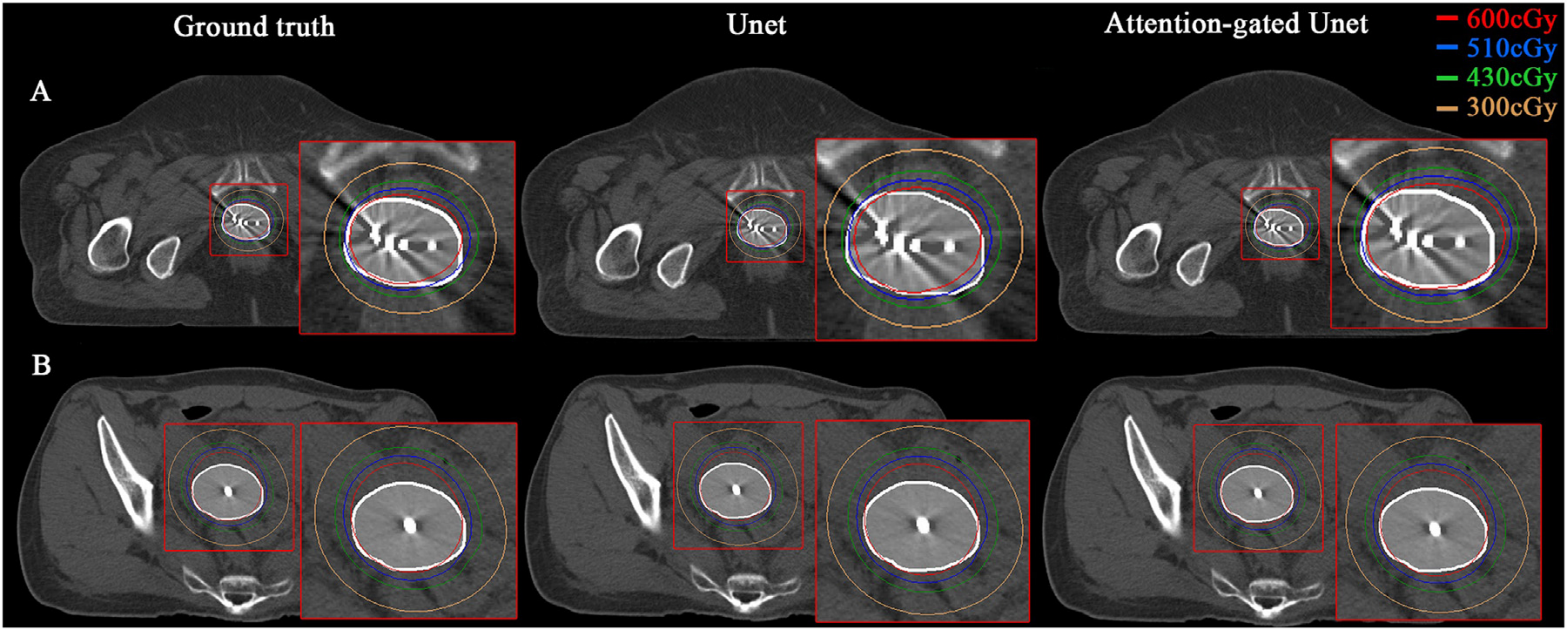
The 3-dimensional (3D) dose distribution of the ground truth (manual digitization), 3D convolutional neural network (CNN), and 3D CNN plus attention gates–based automatic digitization of 2 patient examples. The white shadow area indicates the high-risk clinical target volume.

The dose difference in the CTV and OARs of manual and automatic digitization is shown in Fig. E2. The 3D dose distribution of 2 examples is shown in Fig. 3, where isodose lines generated by the ground truth (manual digitization), CNN, and CNN + AG models were compared in the axial plane. Both the CNN and CNN + AG methods had consistent dose distribution with the manual digitization of interstitial needles (Table E1), which meant the CNN + AG method could be a surrogate for manual digitization of interstitial needles. Also, the CNN + AG method demonstrated slightly better performance than the CNN method. The manual digitization process of HDR brachytherapy is time consuming and can take up to 15 minutes. The entire automatic needle digitization without additional human guidance and rewriting the treatment plan into the commercial TPS takes about 1 minute on average, which reduces the time for needle digitization by 93%. Consequently, the proposed CNN + AG–based automatic digitization method would be integrated into the TPS and used to create clinically acceptable plans for further time savings and would reduce user dependency in HDR brachytherapy treatment planning.

## Discussion

Needle digitization is one of the most critical steps of CT-based HDR brachytherapy planning and remarkably affects planning quality and curative effect.^29^ We have proposed a novel deep-learning method with AGs for automatic digitization to accelerate the workflow of brachytherapy and to avoid potential human errors. Two different 3D Unet–based deep-learning models were created, with and without spatial AGs. Both models had geometric and dosimetric consistency with manual digitization in the TPS. Furthermore, the AGs integrated into the 3D Unet model served as a feature selector by progressively highlighting salient features while suppressing task-irrelevant information. We adopted group normalization to improve the accuracy and convergence speed of automatic needle digitization in this work.

**Figure 4:**
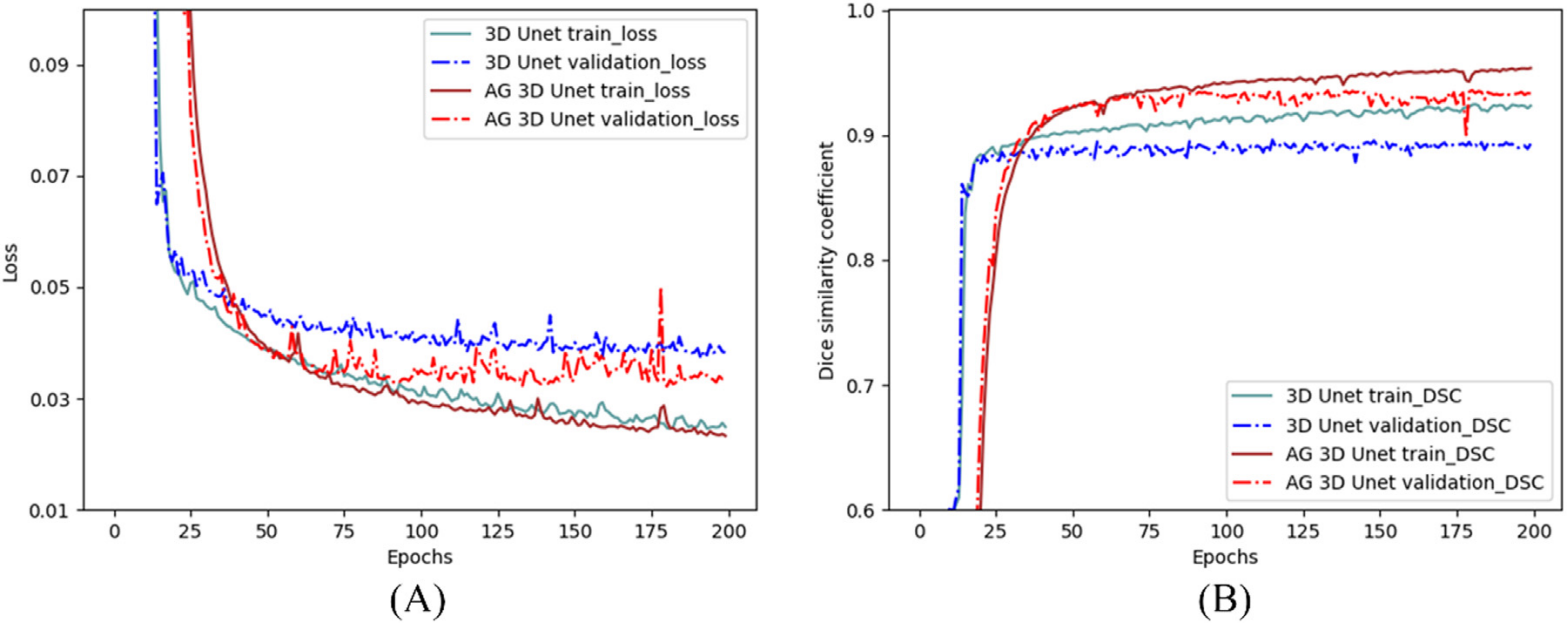
The loss and Dice evolution of the training and validation set.

The results also showed that the performance of the CNN + AG method was superior to the CNN model, with statistically significant improvement in DSC and JI (*P* < .05). In addition, the lowest loss and highest DSC were more quickly reached for the CNN + AG model, showing a faster convergence of the CNN + AG method (Fig. 4).

To the best of our knowledge, this work is the first to report both geometric and dosimetric differences between manual and automatic digitization of interstitial needles in HDR brachytherapy using attention mechanisms. Previously, Hu et al^11^ and Deufel et al^6^ conducted dosimetric analysis using automatic digitization of Fletcher applicators.

There are several limitations in our study. First, the performance of the CNN + AG model was constrained by the slice thickness of CT simulation. Deufel et al^6^ suggested that geometric agreement of manual and automatic digitization could be improved by higher resolution and thinner-sliced CT images. Qing et al^30^ reported that the tip position of needles was affected by the slice thickness of the CT. Hu et al^11^ reported a greater DSC, lower HD95 distance, and smaller tip error for automatic digitization of the Fletcher applicator when CT slice thickness was reduced to 1.3 mm. In the future, CT slice thickness should be reduced to improve accuracy of automatic needle digitization.

Second, we constructed 3D deep-learning models with and without AGs based on CT only and did not include MRI-guided HDR brachytherapy. Magnetic resonance scans should be introduced as input images in the future, with automatic digitization of MRI-compatible needles. Shaaer et al^31^ proposed a 2D Unet automatic reconstruction algorithm based on T1- and T2-weighted MRI, which had potential to replace conventional manual catheter reconstruction, although the reconstruction time was still relatively long (approximately 11 minutes).

Third, potential uncertainty in the manual contour still exists and may affect the accuracy of needle digitization, which could be resolved by implementing more cases to the deep-learning model or inviting multiple planners for the manual-digitization step. Future work will be involved with an end-to-end design for automatic digitization of the HR-CTV, OARs, and needle trajectories with high-resolution CT to improve the overall performance.

## Conclusions

A deep-learning model incorporated with AGs was proposed and evaluated geometrically and dosimetrically for automatic digitization of interstitial needles in HDR brachytherapy for cervical cancer. This model has clinical potential to improve planning efficiency.

## Appendix Supplementary materials

supplementary materials Image, application 1

## Footnotes

Supplementary material associated with this article can be found, in the online version, at doi:10.1016/j.adro.2023.101340.
