# Supplementary material for: Attention-Gated Deep-Learning–Based Automatic Digitization of Interstitial Needles in High-Dose-Rate Brachytherapy for Cervical Cancer
Source: Adv Radiat Oncol. 2023 Aug 10;9(1):101340. doi: 10.1016/j.adro.2023.101340 (PMC10801665; doi:10.1016/j.adro.2023.101340)
Supplement: supplementary materials [file mmc1.docx]

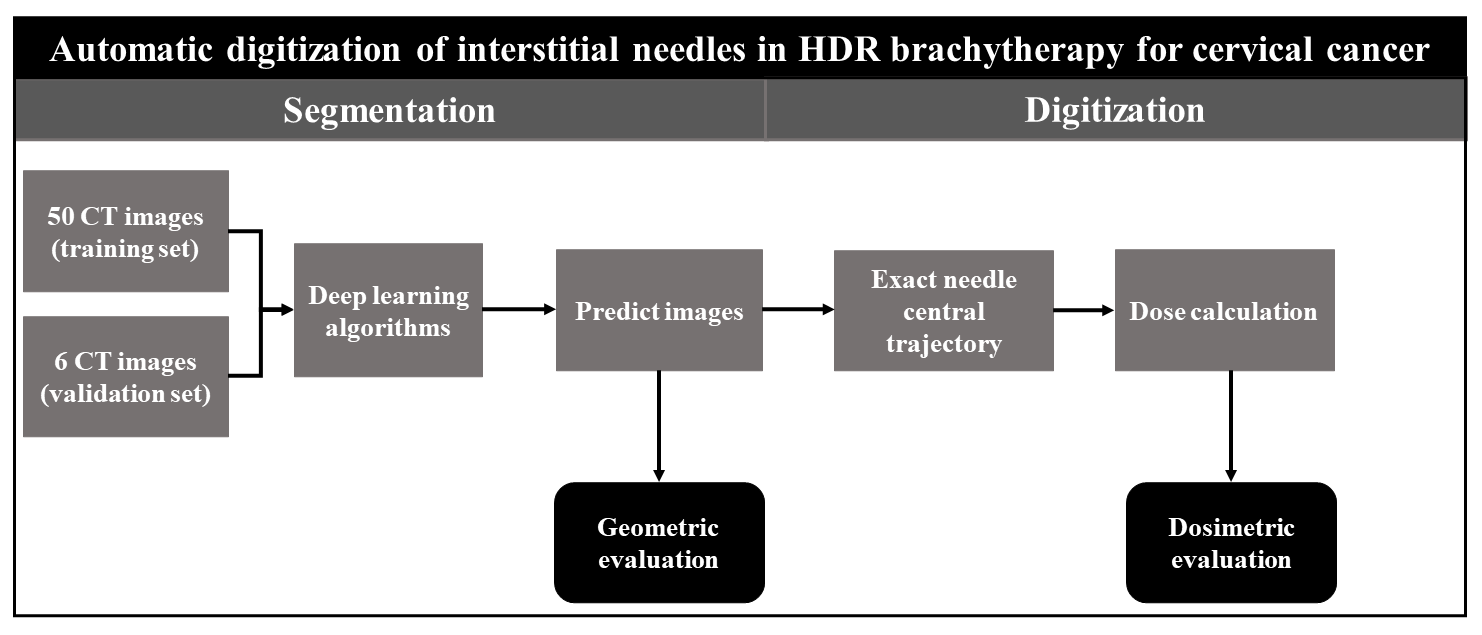


Supplementary Fig.1 (SF1) The workflow of automatic digitization of interstitial needles in HDR brachytherapy.


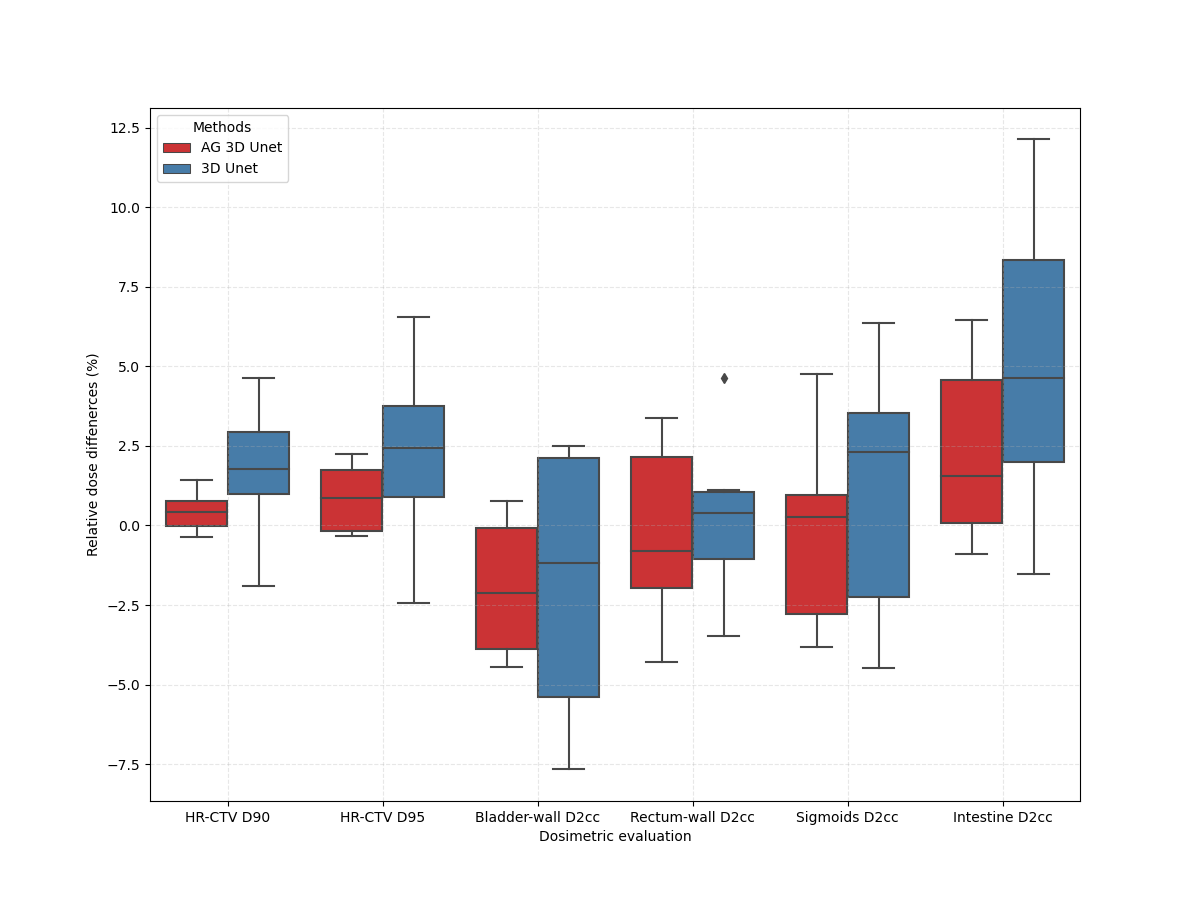


Supplementary Fig.2. Relative dose difference (%) between manual digitized plans and automatic digitized plans in both CTV and OARs.

**Table.S1. Dosimetric differences between manual and automatic reconstructions**

|  | **Dosimetric parameters** | **Mean dose difference (cGy)** | | **Relative dose difference (%)** | |
| --- | --- | --- | --- | --- | --- |
|  |  | 3D Unet | Attention-gated 3D Unet | 3D Unet | Attention-gated 3D Unet |
| **HR-CTV** | D90% | 10.4 ± 12.3 | 2.6 ± 3.6 | 1.7 ± 2.0 | 0.4 ± 0.6 |
|  | D95% | 12.4 ± 15.3 | 4.8 ± 5.7 | 2.3 ± 2.8 | 0.9 ± 1.0 |
| **Bladder wall** | D2cc | -7.8 ± 18.5 | -8.6 ± 9.4 | -1.9 ± 4.2 | -2.0 ± 2.1 |
| **Rectum wall** | D2cc | 0.4 ± 11.1 | -1.5 ± 11.9 | 0.3 ± 2.5 | -0.3 ± 2.8 |
| **Sigmoids** | D2cc | -1.2 ± 11.1 | -3.0 ± 8.4 | 1.1 ± 3.9 | -0.2 ± 2.9 |
| **Intestines** | D2cc | 12.3 ± 13.8 | 9.2 ± 9.8 | 5.1 ± 4.6 | 2.3 ± 2.8 |
